# Redefining resection margins and dissection planes in perihilar cholangiocarcinoma—radical resection is a rare event

**DOI:** 10.1007/s00428-021-03231-1

**Published:** 2021-11-16

**Authors:** Melroy A D’Souza, Hasan Ahmad Al-Saffar, Carlos Fernández Moro, Sonia Shtembari, Olof Danielsson, Ernesto Sparrelid, Christian Sturesson

**Affiliations:** 1 Division of Surgery, Department of Clinical Science, Intervention and Technology (CLINTEC), Karolinska Institute, Karolinska University Hospital, 14186 Stockholm, Sweden; 2 Department of Clinical Pathology and Cytology, Karolinska University Hospital, Stockholm, Sweden; 3 Division of Pathology, Department of Laboratory Medicine (LABMED), Karolinska Institute, Stockholm, Sweden

**Keywords:** Perihilar cholangiocarcinoma, Margin status, Klatskin tumor, Resection margins

## Abstract

Radical tumor resection (pR0) is prognostic for disease-free and overall survival after resection of perihilar cholangiocarcinoma (pCCA). However, no universal agreement exists on the definition of radical resection and histopathological reporting. The aim of this study was to provide a standardized protocol for histopathological assessment and reporting of the surgical specimen obtained after resection for pCCA. All consecutive patients operated for pCCA with curative intent at the Karolinska University Hospital, Stockholm, Sweden between 2012 and 2021 were included. A standardized protocol for histopathological assessment and reporting of the surgical specimen after liver resection for pCCA is presented. A detailed mapping of the transection margins and dissection planes was performed. The results of applying different existing pR0 definitions were compared. Sixty-eight patients with pCCA were included. Five transection margins and two dissection planes were defined. By defining pR0 as cancer-free margins and planes tolerating distances <1mm, the pR0 rate was 66%. However, when pR0 was set as >1mm from invasive cancer to all resection margins and dissection planes, the pR0 rate fell to 16%. This study supports the use of thorough and standardized pathological handling, assessment and reporting of resection margins and dissection planes of surgical specimens of pCCA.

## Introduction

Perihilar cholangiocarcinoma (pCCA) arises from the biliary epithelium of the hepatic hilum and even when treated with curative intent is generally associated with poor outcomes [1–3]. Curative surgery has become more aggressive and commonly involves a major hepatectomy, resection of the extrahepatic bile ducts and a complete hepatoduodenal ligament lymphadenectomy and vascular resection unconditionally or ‘on demand’[4]. Radical tumor resection (pR0 resection) has proven prognostic for disease-free and overall survival [5, 6]. However, there is no universal consensus on which resection margins should be included in the pathological examination of the surgical specimen to determine and report on the final status of the resection margin (pR0/pR1) [7, 8]. Resection margins include both ‘transection margins’ where structures are divided or transected and ‘dissection planes’ where structures are surgically freed or dissected from their surrounding anatomy. Bile duct margins are an example of ‘longitudinal’ transection margins and the ‘radial’ or ‘circumferential’ margin in the hepatoduodenal ligament, that of a dissection plane. These parameters have been described varyingly by different centers with bile duct margins being the most commonly reported in the literature [5, 8–10].

In addition, there is no universal agreement on the definition of what constitutes a ‘pR0’ resection in pCCA and how wide the tumor-free margin should be [11]. Differing definitions have been described with 1 mm as the usual cutoff value for a tumor-free margin [7, 11]. The International Collaboration on Cancer Reporting (ICCR) published a consensus guideline for pathology reporting in cholangiocarcinoma and defined pR0 as tumor-free margin of ≥ 1 mm [12, 13].

As the concept of pR0 resection carries profound prognostic value, it is of importance to have a common universal definition of ‘pR0’ when comparing results from different institutions and when designing studies factoring in the surgical treatment of pCCA. The aim of this study was to provide a systematic and standardized protocol for orientation, inking, sampling, histopathological assessment and reporting of the surgical specimen obtained after liver resection for pCCA. A detailed mapping of the transection margins and dissection planes was performed. Based on this investigation on the surgical specimens from a single center, the results of applying different existing R0 definitions are compared.

## Patients and methods

### Patients

All consecutive patients operated for pCCA with a major liver resection (≥3 Couinaud’s segments) with curative intent at the Karolinska University Hospital, Stockholm, Sweden between 2012 and 2021 were included in the study. pCCA was defined as a tumor originating above the junction of the cystic duct with the common hepatic duct and up to and including the second-order biliary branches [13]. All patients were discussed at a multidisciplinary team conference before scheduled for surgical treatment. The study protocol was approved by the regional ethical committee in Stockholm, Sweden in accordance with the guidelines of the Helsinki Declaration (2015/259-31/2).

### Surgical procedure

In case of jaundice, patients underwent biliary decompression, preferably via the endoscopic route, but the percutaneous approach was used when deemed appropriate. The type of liver resection was decided based upon the preoperative morphological and functional assessment. If the calculated future liver remnant was <30% of the total liver volume, portal vein embolization was performed, including the portal branches of segment 4 in case of a planned right trisectionectomy. The operation was performed via a right-sided subcostal incision. The aortocaval (station 16b) and hepatic artery lymph nodes (station 8a) were sampled. The retropancreatic lymph nodes (station 13) were sampled at the discretion of the operating surgeon. If the liver artery to the future liver remnant was found free from tumor invasion, the distal bile duct was cut just cranial to the pancreas, and the transection margin sent for frozen section analysis. In case of cancer-positive margin, a combined pancreatic resection was considered. The transected bile duct was then reflected cranially skeletonizing the portal vein by including all the periductal soft tissue and lymph nodes ‘en bloc’ with the surgical specimen. The portal vein and artery to the hemi-liver to be resected were divided at their origin. In case of intraoperative suspicion of tumor engagement of the portal vein, portal vein resection was performed as a final measure. Liver parenchymal transection was performed using the Cavitron Ultrasonic Surgical Aspirator (CUSA). In general, the caudate lobe of the liver (segment 1) was included in the resection. Marking of the different margins was not systematically performed by the operating surgeon.

### Pathology procedure

**Fig. 1:**
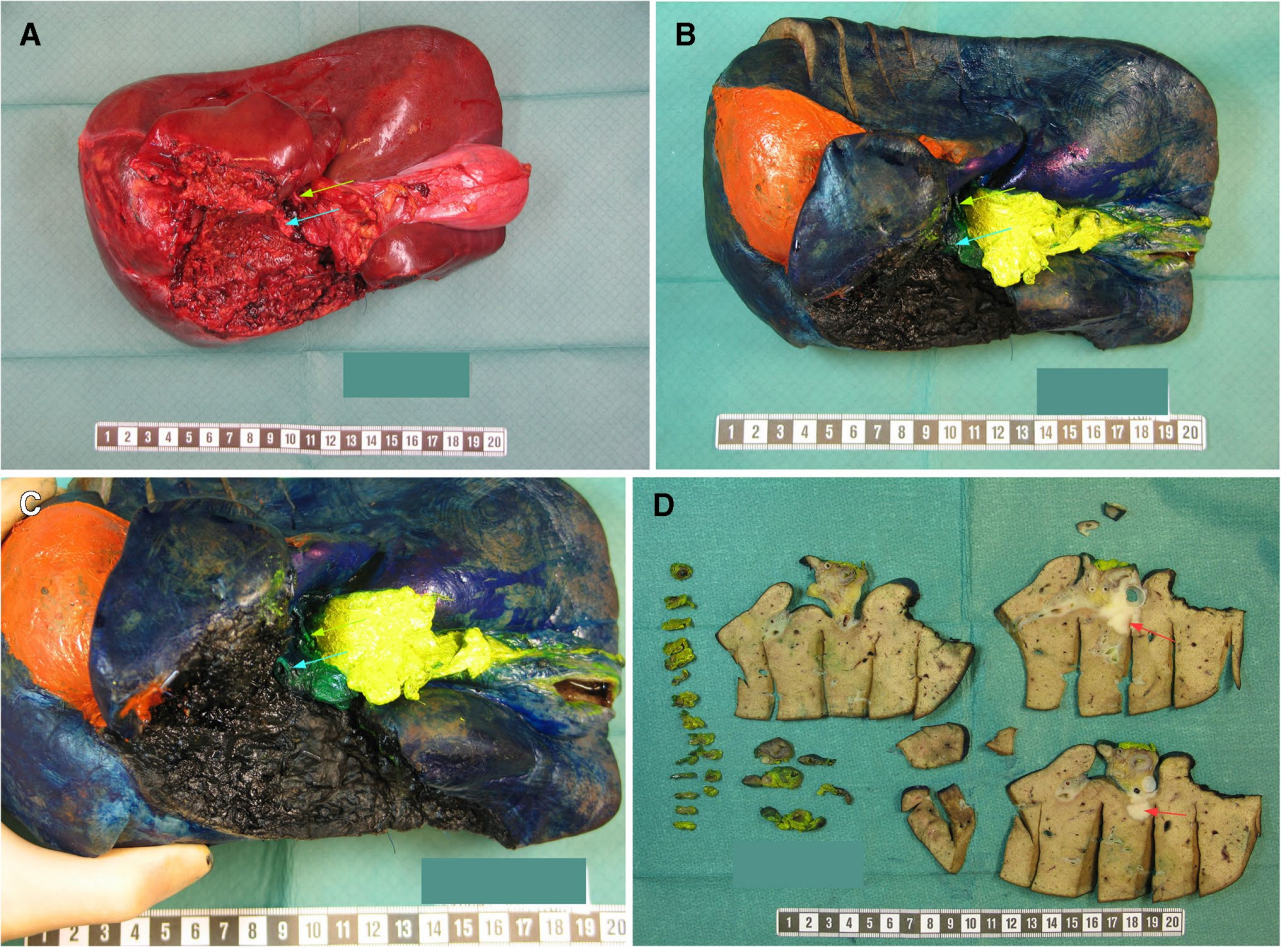
Macroscopic assessment of surgical specimen for pCCC. **A** Fresh view of the surgical resection specimen immediately after the operation. **B** Formalin-fixed specimen after inking of the different resection margins: hilar—green, hepatoduodenal ligament—yellow, hepatic parenchyma—black, coronal ligament—orange. The peritoneal surface (not a resection margin) is inked in blue. **C** Close-up view of the hilar region showing the stumps and resection margins of the proximal bile duct and the portal vein. **A**–**C** Green arrows indicate portal vein and blue arrows bile duct stumps-resection margins, respectively. **D** Photographic overview after serial slicing of the formalin-fixed specimen along the common bile duct and hepatoduodenal ligament (yellow inked) and the liver at the hilar region. A pCCC is seen as a solid mass (red arrows) extending between the bile duct and the adjacent liver, which is invaded

The resected specimen was sent fresh on ice to the pathology laboratory where all examinations and processing were carried out by a specialized hepatobiliary pathologist. The specimen was oriented in the craniocaudal and mediolateral aspects and photographed on its anterior and posterior surfaces as well as the hilar region (Fig. 1A).

The different surfaces, transection margins and dissection planes were identified and inked following a standardized color scheme (Figure 1B,C). The visceral peritoneum (in blue) is recognized by its smooth, glistening surface covering the liver and the ventral aspect of the hepatoduodenal ligament. The *hilar dissection plane* (in green) is a concave or cuneiform region of smooth, matte surface containing the stumps and transection margins of the proximal hepatic bile duct and the portal vein; it is limited by the visceral peritoneum, the hepatic transection margin and the insertion of the soft tissue sheath of the hepatoduodenal ligament. The *hepatoduodenal ligament dissection plane* (in yellow) has a mostly smooth, matte surface with a quality of soft tissue fascia although, especially in its lateral and caudal ends, may present coarser areas of transected fat tissue; this plane defines the posterior, retroperitoneal surface of the soft tissue sheath that embeds the common bile duct and is limited proximally by the hilar dissection plane, which is devoid of fat tissue, and distally by periductal soft tissue around the transection margin of the common bile duct. The *proximal and distal bile duct transection margins* (of the hepatic bile duct and the common bile duct respectively) are found in the hilar region and the distal end of the soft tissue sheath of the hepatoduodenal ligament, respectively. Probing the bile duct was discouraged, as it may damage or detach an eventual intraductal tumor component. The *transection margin of the portal vein* is located in the hilar region, close to the entrance of the vessel into the resected liver and was identified most often by the presence of metal staples. The *transection margin of the hepatic artery* is usually identified more distally, embedded in the soft tissue of the hepatoduodenal ligament and ligated by suture-ligature close to its end. The *hepatic parenchymal transection margin* was inked in black. In addition, all other non-peritonealized surfaces, which also constitute circumferential resection margins, like the transection margin in the teres ligament and the retroperitoneal dissection planes in the coronary ligament and the left triangular ligament, as well as resected planes of adherence in the visceral peritoneum, for example with portions of the omentum, were also inked (in orange).

After inking, the resection margins were covered with absorbent paper and sprayed with acetic acid to fixate the stains and avoid blending of the colors. Afterwards, parallel axial cuts were performed every 1.5 cm along the anterior surface of the liver without cutting it completely, and a double sheet of absorbent paper was placed in each fold, to improve the penetration and fixation of the tissue by the formalin. The specimen was then wrapped in gauze to make a bundle and tied with safety pins to prevent tissue deformation and preserve the original anatomical configuration. Finally, the specimen was rested in formalin for at least 96 h to allow an adequate tissue fixation.

After fixation, ‘grossing’ of the specimen was performed according to a standardized protocol. Firstly, the intact inked specimen was photographed, the dimensions of the resected anatomical structures measured and the total weight recorded. The hepatic artery transection margin was first sampled followed by the distal bile duct transection margin. Then, the soft tissue sheath of the hepatoduodenal ligament containing the embedded common bile duct was cut loose from the specimen close to its proximal insertion near the hilar plane and cut serially in transverse sections from the distal end into 3-mm-thick tissue slices. Next, the proximal bile duct transection margin and the portal vein transection margin were identified in the hilar region and sampled. Finally, the liver, including the gallbladder when present, was sliced serially in the axial plane starting from the hilar region into 3–4-mm-thick slices. The tissue slices and sampled transection margins were subsequently laid in order and oriented in a tray and photographed, both in overview and detail (Fig. 1D). The overview picture was printed out for subsequent topographical documentation of tissue sampling for histology.

After tissue processing for histology, the pathologist examined the slides under the microscope, keeping track of their anatomical location in the photographic overview, and reported the cancer according to a standardized structured pathology protocol consistent with the guidelines published by the International Collaboration on Cancer Reporting (ICCR) [13]. This included a precise identification of tumor origin according to AJCC 8^th^ edition, a detailed mapping of tumor extension into neighboring structures, a comprehensive assessment of the different resection margins, pathways of tumor propagation (lymphovascular, perineural), status of the visceral peritoneum, lymph nodes and immunohistochemical investigations.

**Fig. 2:**
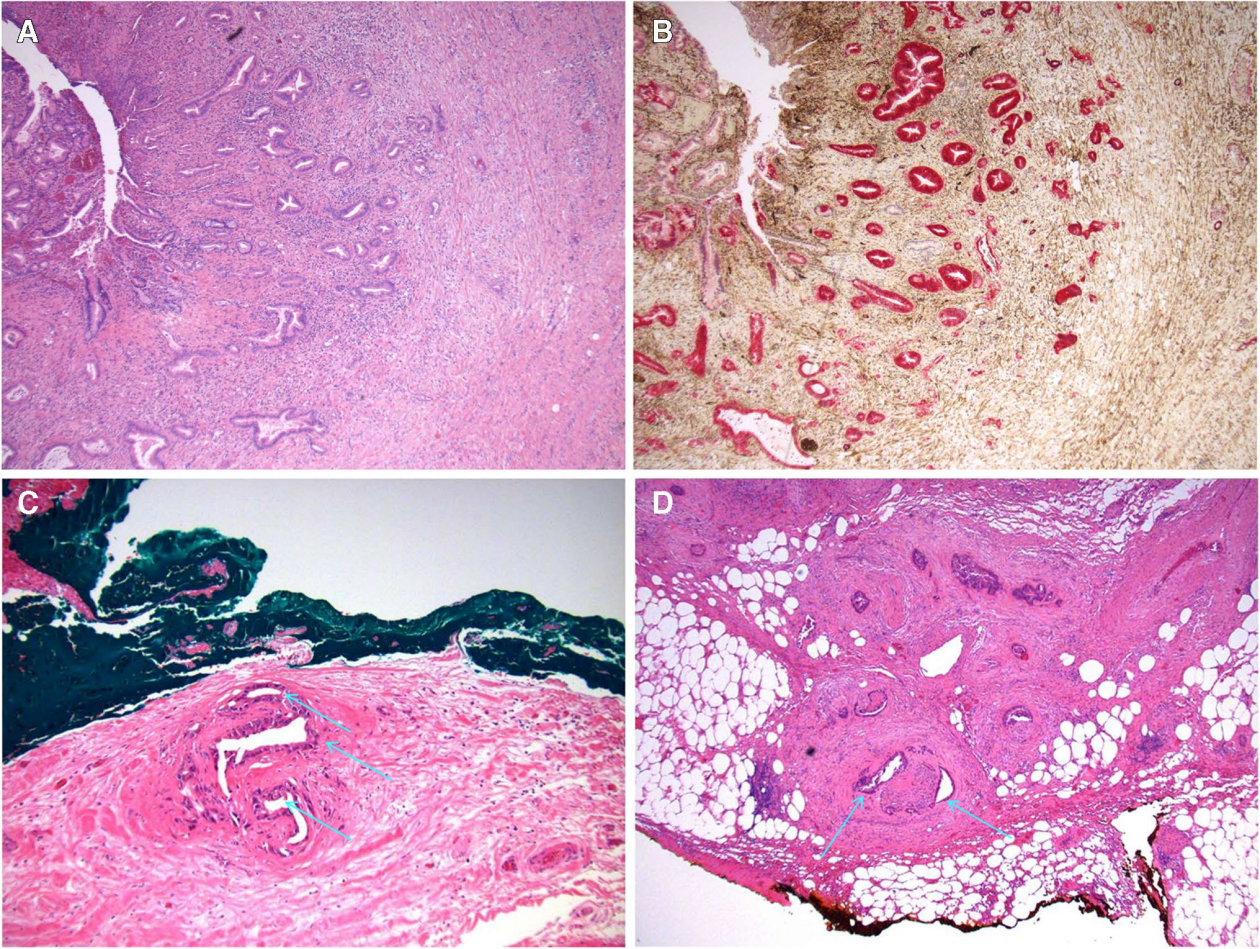
Microscopic assessment. **A**, **B** Infiltrate of pCCC involving the bile duct (**A**: hematoxylin and eosin, 4×; **B**: immunohistochemistry, 4×, where cancer cells stain strongly for maspin/red). **C** Hilar (10×) and (**D**) hepatoduodenal ligament (4×) resection margins in surgical specimen of pCCC. Tumor cells representing perineural growth are present < 1 mm (arrows) from the inked resection margin

The resection margins were assessed and reported according to a specified criteria and scheme. Positive resection margins were defined as: presence of tumor cells in the histological section of the following transection margins: proximal bile duct, distal bile duct, portal vein and hepatic artery; or presence of tumor within a distance of less than 1 mm to any of the following margins: hilar and hepatoduodenal ligament dissection planes and hepatic parenchymal transection margin. Otherwise, as a minimum, the distance to closest margin was reported. Recommendation was in any case made to systematically report the minimal distance (in mm) to all margins, with exception of the vascular ones (hepatic artery and portal vein). Five transection margins and two dissection planes were reported. Examples of positive hilar and hepatoduodenal ligament dissection planes are shown in Fig. 2A,B, respectively.

The global margin status was finally regarded as pR1 if any resection margin was positive according to the criteria above; or as pR0 when all were negative for tumor.

Summary statistics were presented as whole numbers and percentages for categorical variables, or as medians with interquartile range for continuous variables. Kaplan–Meier analysis was used to estimate survival from the time of operation, and the log rank test was used for testing equality of survival functions between groups.

## Results

In total, 68 patients with pCCA were included in the study. Patient characteristics and procedure-related data are shown in Table 1 including perioperative variables, tumor classification according to Bismuth-Corlette and surgical procedure details. Table 2 shows the minimal distance from invasive cancer to the different resection margins. Only one patient had a positive hepatic artery margin in the cohort. For the 17 patients subjected to portal vein resection and anastomosis in the liver hilum, 3 patients had cancer present in the dissection planes at the liver hilum, 11 patients had <1mm margin to invasive cancer, while 3 patients had a margin of >1mm. By defining pR0 as absolute cancer-free margins tolerating distances <1mm, the pR0 rate was 66%. However, when pR0 was set as >1mm from invasive cancer to all resection margins, the pR0 rate fell to 16%.

**Table 1:** Patient and tumor characteristics

Patient, tumor and perioperative characteristics	Perihilar cholangiocarcinoma (*n*=68)
Age (years)	62 (52–70)
Males/females	39/29
Preoperative biliary decompression (%)	53 (78 %)
Preoperative portal vein embolization (%)	23 (34 %)
Preoperative serum bilirubin level (µmol/l)	15 (10–26)
Preoperative serum albumin level g/l	32 (28–36)
Bismuth Corlette classification	
Bismuth 1 (*n*)	2
Bismuth 2 (*n*)	6
Bismuth 3a (*n*)	41
Bismuth 3b (*n*)	13
Bismuth 4 (*n*)	6
Surgical characteristics	
Extended right hemihepatectomy	38 (56%)
Extended left hemihepatectomy	9 (13%)
Left hemihepatectomy	13 (19%)
Right hemihepatectomy	8 (12%)
Concomitant resection of segment 1	56 (82%)
Portal vein resection and reconstruction	17 (25%)
Concomitant pancreatic resection	4 (6%)

**Table 2:** Resection margin assessment in perihilar cholangiocarcinoma

Minimal distance (*d*) from invasive cancer to resection margin	Proximal bile duct transection margin	Hepatic transection margin	Hilar dissection plane	Hepatoduodenal ligament dissection plane	Distal bile duct transection margin	Hepatic artery transection margin	Portal vein transection margin
*d*=0 mm or present in transection margin	8 (11%)	2 (3%)	8 (12%)	6 (9%)	5 (7%)	1 (1%)	0
*d*≤1 mm	9 (13%)	9 (13%)	39 (57%)	27 (40%)	0 (0%)	-	-
*d*>1 mm	52 (76%)	57 (84%)	21 (31%)	35 (51%)	63 (93%)	-	-

Median follow-up time after surgery was 62 (28–82) months. Overall survival of patients from the time of operation, excluding six patients (9%) who died within 90 days postoperatively, was 31 (CI95% 16–46) months, with a 5-year survival of 32%. Recurrence free survival was 22 (CI95% 15–29) months. No significant impact on the chosen definition of R0 (>0 mm or >1 mm to cancer-involved resection margin or dissection plane) was found neither for overall (*P*=0.472, *P*=0.705, respectively) or recurrence free survival (*P*=0.314, *P*=0.155, respectively).

## Discussion

In the present study, a standardized protocol for pathological assessment of the surgical specimen after resection of pCCA is introduced. Five transection margins and two dissection planes are used to evaluate surgical radicality of the specimen in the pathological examination of resected pCCA and take into consideration the surgical procedure performed for pCCA as the conceptual basis for their use in reporting.

A hilar dissection plane ventral to the portal bifurcation is defined, which corresponds largely to the circumferential margin at the level of the Glissonian pedicle, that is, around the hepatic duct close to the (right-left) confluence region and containing the resection margin or stump of the portal vein. It is grossly identified by its proximal location at the hepatic hilum and a smooth surface devoid of the periductal fat, which is present more distally along the hepatoduodenal ligament. These features make the hilar dissection plane analogous to the superior mesenteric groove dissection plane as found in pancreaticoduodenectomy specimens. Following the bile duct distally, a sheath of periductal fat belonging to the hepatoduodenal ligament is evident. It is layered anteriorly by the visceral peritoneal, while its dorsal dissection plane, which is just ventral to the main portal vein and the hepatic artery, corresponds to the dissection plane in the hepatoduodenal ligament. These features make it comparable to the posterior retroperitoneal dissection plane in pancreatoduodenectomy specimens. These two dissection planes have recently been included in the definition of circumferential or radial margins after resection of pCCA in a study by Shinohara et al. [5]. The radial or circumferential margins described in the literature correspond to the hilar dissection plane and the dissection plane in the hepatoduodenal ligament as presented in our study. Further subdividing the radial margin into two distinct anatomic regions as introduced here could carry prognostic information. Little importance has previously been given to the radial margin in studies on perihilar cholangiocarcinoma. Shinohara et al. showed that the radial margin was the margin most frequently involved by cancer after resection of pCCA, amounting to 11% of a total of 18% with margin positivity in the cohort [5]. In addition, a cancer-positive radial margin was shown to have a negative effect on survival. Information on radial margin has frequently been missing in pathology reports, as illustrated in the study by Chatelain et al. [9]. In the present study, a cancer-positive hilar dissection plane of 12% and a cancer-positive hepatoduodenal ligament dissection plane of 6% were found. The diverging rates of margin involvement may well reflect a locally advanced tumor in the hilar region and further spread along the hepatoduodenal ligament, which commonly occurs with perineural tumor growth and intravascular spread [14].

It could be argued that a lower rate of margin positivity in the hilar dissection plane could be achieved by a higher usage of portal vein resection and reconstruction. The principle of routine portal vein resection has been advocated by previous investigators [15, 16]. However, without detailed description on the pathology procedure and of which margins are being microscopically investigated, it is difficult to draw conclusions regarding the effect on radicality of such a surgical strategy. In the present series, portal vein resection and reconstruction were only performed when there was an intraoperative suspicion of macroscopic vascular invasion. In total, 17 patients underwent portal resection. However, there were still three patients with a cancer-positive hilar dissection plane, and another 11 patients within this group had cancer cells within a distance of <1 mm in this plane. This could be explained by the physical constraints of the anatomically complex hilar region, which makes it very difficult to safely achieve clearance distances ≥ 1 mm in this particular plane in locally advanced tumors. This can also be seen as analogous to pancreatic head resections for cancer, where it has been shown that resection of the superior mesenteric vein confluence when vessel involvement is suspected does not increase the rate of pR0 resection [17].

The hepatoduodenal ligament dissection plane, as described in the present study, signifies collectively what has previously been named arterial resection plane, portal vein resection plane, radial and periductal dissection planes all together [5, 8]. The surgical technique used in the present study has been an ‘en bloc’ caudal-to-cranial dissection of the hepatoduodenal ligament after first dividing the bile duct at the entrance into the pancreas and dividing the appropriate portal vein and hepatic artery branches when encountered.

The other resection margins assessed in the study include proximal and distal bile duct transection margins, the vascular margins and the hepatic parenchymal transection margin, as routinely described by other investigators. The ‘longitudinal’ transection margins (bile duct and vascular) were embedded and sectioned at the surface opposite to the ‘true’ surgical end at a maximal thickness of 3 mm. This technique prevents eventual loss of tumor-bearing tissue as a result of histological sectioning at the surgical end and improves the quality of histomorphological assessment, as the tissue at the surgical end usually suffers operative trauma artefacts. This also allows the possibility of obtaining additional histological sections toward the ‘true’ surgical end in case of suspicious but non-conclusive findings in the initial histological section. In contrast, the dissection planes and hepatic transection margin were embedded such that the ‘true’, inked surgical margin is always present and readily identifiable in the histological sections, allowing a precise measurement of the distance of tumor cells to the inked margin. While vascular margins were routinely sampled in our study, only one patient had a positive hepatic artery margin in this cohort.

The definition of pR0 varies globally. In pCCA, pR0 is proposed to mean a tumor-free margin of ≥1 mm according to the ICCR [13]. However, many studies used cancer-free margins (>0 mm) to define pR0 [5, 7, 9]. Especially at the hilar dissection plane, the definition used is of great significance. As shown in Table 2, when pR0 is defined by a ≥1-mm margin, the percentage of patients classified as pR0 at the hilar dissection plane was only 31% in the present study. This is due to the anatomical prerequisite of only a thin sheath of connective tissue between the bile duct and surrounding structures in the Glissonian pedicle. It should be emphasized that the peritoneal surfaces are not considered as resection margins, and eventual tumor involvement of the peritoneum should be accounted for separately.

In the present study, extensive, complete or near complete, sampling of the tumor, extrahepatic bile ducts, hepatoduodenal ligament, hilar and perihilar regions was routinely performed, the reasons for this being the poor reliability of the macroscopic assessment in discriminating between tumor and fibrosis. It also helps to precisely map cancer invasion into the neighboring structures (which determines pT-staging) and microscopic cancer spread (per continuum, intravascular, perineural), to thoroughly assess the different resection margins and to map the presence and extent of an eventual (dysplastic) premalignant lesion. During sampling, the pathologist marked out in the printed photographic overview where in the macroscopic tissue slices each tissue piece (block) that was collected for histology came from.

No significant correlation of pR status with survival outcome was found, most likely because of the small number of patients included in the study and its dependence on many other clinical, surgical and pathological factors [6]. This study introduces and supports the use of thorough and standardized pathological handling, assessment and reporting of resection margins of surgical specimens of pCCA.

## Data Availability

All data generated or analyzed during this study are included in this published article.
